# Difference of Excretion of Carbonic Acid and Absorption of Oxygen under Various Circumstances

**Published:** 1869-04

**Authors:** 


### Selections.

DIFFERENCE OF EXCRETION OF CARBONIC ACID AND ABSORPTION OF OXYGEN UNDER VARIOUS CIRCUMSTANCES.—Pettenkofer and Voit have presented to the Academy of Munich a communication on the difference of the excretion in the human subject, of carbonic acid and absorption of oxygen by day and night, during rest and labor. The new arrangement of their great respiratory apparatus easily admits of a division of 24 hours' investigation into two periods.

The subject for observation, a sound, strong watchmaker 28 years of age, and of 60 kilogrammes weight, entered the apparatus July 31, at 6 A. M., and remained in it till August 1, 6 A. M. The time from 6 A. M. till 6 P. M. is understood to be day, the rest night. The man took, at his regular meal hours, medium quantities of his usual food whose elementary composition had been carefully made out. He occupied himself in reading and in cleaning a little watch; went to bed at 8 P. M. and slept well till 5 A. M. His condition was perfectly normal during the investigation.

31ST JULY, 1866. DAY OF REST.

The numbers express grammes (500 grammes a pound.)

| | Excrated. | | | |
|---|---|---|---|---|
| Period. | Carbonic Acid. | Water. | Urea. | Absorbed Oxygen. |
| Day, | 532.9 | 344.5 | 21.7 | 234.6 |
| Night, | 378.6 | 483.5 | 15.5 | 474.3 |
| Total, | 911.5 | 820.0 | 37.2 | 708.9 |

August 3, at 6 A. M., the same man entered the apparatus again to remain in it 24 hours, but doing labor during day time. He had to turn a wheel loaded with 25 kilo. (50 lbs.), and made, with interruptions for meals and rest, as usual with laborers, 7,323 turns. At 5½ P. M., when he had done, he felt tired as after a day of hard work or after a long march. The food was the same as on July 31, also the time of meals. He took only 600 grammes more water, which, on both days, he was allowed to drink at liberty. After supper he soon went to rest and slept till 5 A. M., when he awoke with new vigor.

3D AUGUST. DAY OF LABOR.

| | Excreted. | | | |
|---|---|---|---|---|
| Period. | Carbonic Acid. | Water. | Urea. | Absorbed Oxygen. |
| Day, | 884.6 | 1094.8 | 20.2 | 294.8 |
| Night, | 399.6 | 947.3 | 16.9 | 659.7 |
| Total, | 1284.2 | 2042.1 | 37.0 | 954 5 |

As will be seen, the excretion of carbonic acid amounted during day time to 58 per cent., during night to 42 per cent., the absorption during day time to only 33 per cent., during night to 67 per cent. The excretion of urea, as has been known heretofore, is always greater during day than during night.

On the day of rest, the excretion of urea, in both daily periods, was in exact proportion to the excretion of carbonic acid; there was excreted of both 58 per cent. during day and 42 per cent. during night. Most surprising is the antagonism in the excretion of carbonic acid and the absorption of oxygen between the two daily periods, even with all possible avoidance of muscular exertion, on July 31. This shows that mere waking and the impressions experienced by the senses in that state, have an influence on the consumption of material serving to produce the processes of life.

This antagonism is still greater when the day of labor is included into the comparison; the excretion of carbonic acid and the absorbtion of oxygen by day and night, are then in exactly inverse proportion. The carbonic acid was excreted at the rate of 69 per cent during day, 31 per cent. during night; the oxygen absorbed at the rate of 31 per cent. during day, 69 per cent during night.

For day time, while awake we therefore produce a great deal of carbonic acid at the expense of the oxygen absorbed during rest and sleep. The will finds the material prepared for its spontaneous movements.

So much oxygen as is used up in excess during one day, so much is taken up for compensation during the following night, and as long as this takes place, the body is prepared in the morning for new labor. In comparing the total of the two days of experiment, it appears that, on the day of labor, there were 373 grammes of carbonic acid more excreted than on the day of rest, and 246 grammes of oxygen more absorbed—373 grammes of carbonic acid containing 271 grammes of oxygen, there is only a difference of 25 grammes of oxygen used in excess of the amount taken up from the air.

It is remarkable that on August 3, during labor, there was no notable increase in the absorption of oxygen over that on July 31 during rest, viz: 43 grammes. The authors conclude from this that it can not be the want of oxygen which, in bodily exertion, compels to more frequent and deeper inspirations, but that it is the want of getting rid of the surplus of carbonc acid produced, and of moderating the heat of the blood. No less remarkable is the parallelism of the excretion of carbonic acid, during night, with the absorption of oxygen during day. There is no great difference in the absorption of oxygen during day—the individual may be engaged in bodily labor or may rest; and likewise there is no noticeable difference in the excretion of carbonic acid during night—the person may, on the preceding day, have exerted himself bodily or not. It is certainly not by accident that the oxygen excreted with the carbonic acid of the night after rest, as well as after labor, approaches very nearly the amount of oxygen taken up from the air on the preceding day.

From the result of the excretion of water it appears that, after a fatiguing labor, a man not only gets momentarily into perspiration, but perspires the following night more than after a day of quiet.

The investigations show positively that the absorbed oxygen is never directly used for oxidation down to the last products of combustion, but that there are intermediate stages of oxidation by which the oxygen is retained in the body for hours before it reappears in the form of carbonic acid and water, a fact which previous experiments on marmots during winter sleeps have demonstrated.

The discovery of Voit, made six years ago on the dog, that with the greatest muscular exertion there is not more albumen decomposed than with perfect rest, has been confirmed by the experiments of July 31 and August 3, made on man. On both days, so different in regard to muscular exertion, there was not more nitrogen excreted through the kidneys and bowels than an amount contained in the food that had been taken. There is, however, an intimate connection between the amount of the albumen of the food and the exhibition of spontaneous muscular force. Experiments of Henneberg indicate that the poorer the food in albumen, the less the stock of oxygen that can be accumulated during night, so that more oxygen has to be taken up in day time, even when the whole consumption in the 24 hours is less.—*Med. Repertory.*

